# Effects of Occult Hypoperfusion on Local Circulation and Inflammation - An Analysis in a Standardized Polytrauma Model

**DOI:** 10.3389/fimmu.2022.894270

**Published:** 2022-06-21

**Authors:** Sascha Halvachizadeh, Yannik Kalbas, Michel Paul Johan Teuben, Henrik Teuber, Nikola Cesarovic, Miriam Weisskopf, Paolo Cinelli, Hans-Christoph Pape, Roman Pfeifer

**Affiliations:** ^1^ Department of Trauma, University Hospital Zurich, Zurich, Switzerland; ^2^ Harald Tscherne Research Laboratory, University of Zurich, Zurich, Switzerland; ^3^ Department of Health Sciences and Technology, Eidgenössische Technische Hochschule (ETH) Zurich, Zurich, Switzerland; ^4^ Center for Surgical Research, University Hospital Zurich, University Zurich, Zurich, Switzerland

**Keywords:** local inflammation, porcine model, standardized polytrauma, occult hypoperfusion, persistent lactic acidosis, treat, perfusion in polytrauma

## Abstract

**Introduction:**

Occult hypoperfusion (OH) is defined as persistent lactic acidosis despite normalization of vital parameters following trauma. The aim of this study was to analyze the association of occult hypoperfusion with local circulation and inflammation of injured soft tissue in a porcine polytrauma model.

**Methods:**

This experimental study was performed with male landrace pigs who suffered a standardized polytrauma, including a femoral fracture, blunt chest trauma, liver laceration and a mean arterial pressure (MAP) controlled hemorrhagic shock. One hour after induction of trauma, the animals were resuscitated with retrograde femoral nailing, liver packing and volume replacement. Animals were stratified into Group Norm (normalizing lactate levels after resuscitation) and Group occult hypoperfusion (OH) (persistent lactate levels above 2 mmol/l with normalizing vital parameters after resuscitation). Local circulation (oxygen saturation, hemoglobin amount, blood flow) was measured with optical sensors at the subcutaneous soft tissue at the fractured extremity as well as at the stomach and colon. Local inflammatory parameters [interleukin (IL) 6, 8, 10, and heat shock protein (HSP)] were analyzed in the subcutaneous tissue of the fractured extremity.

**Results:**

Group Norm (n = 19) and Group OH (n = 5) were comparable in baseline vital and laboratory parameters. The shock severity and total amount of blood loss were comparable among Group Norm and Group OH. Following resuscitation Group OH had significantly lower local relative hemoglobin amount at the injured soft tissue of the fractured extremity when compared with Group Norm (39.4, SD 5.3 vs. 63.9, SD 27.6 A.U., p = 0.031). The local oxygenation was significantly lower in Group OH compared to Group Norm (60.4, SD 4.6 vs. 75.8, SD 12.8, p = 0.049). Local IL-6 in the fatty tissue was significantly higher in Group OH (318.3, SD 326.6 [pg/ml]) when compared with Group Norm (73.9,SD 96.3[pg/ml], p = 0.03). The local circulation at the abdominal organs was comparable in both groups.

**Conclusion:**

OH is associated with decreased local circulation and increased local inflammation at the injured soft tissue of the extremity in polytrauma. OH might reflect the severity of local soft tissue injuries, and guide treatment strategies.

## Introduction

The initial assessment and surgical strategy in the treatment of polytrauma patients is based on the physiologic reaction to injuries (1). The early polytrauma management focuses on the stabilization of the triad of acidosis, coagulopathy, and hypothermia. This triad, combined with the soft tissue damage are prominent predictors for mortality in polytrauma patients (1). The physiologic stabilization of the patient represents one guide of the surgical strategy after polytrauma (2). Despite standardized and improved resuscitation, selected polytrauma cases present with persistent lactic acidosis even after responding to resuscitation. The presence of elevated lactate levels during the clinical course has been interpreted as a sign of occult hypoperfusion (OH). OH in trauma is associated with increased risk for infections and other complications (3). The outcome of trauma patients improves if OH is detected and treated early (4). Several different definitions of OH exist, most of which describe a persistent elevated lactate level with concomitant normal vital parameters (3, 5). Risk factors for the development of OH include Injury Severity Score (ISS), Glasgow Coma Scale (GCS) at admission, hypotension, and advanced age (4). OH has only been approximated by systemic measures or analysis of CO2-gaps between central veins and arteries (6). In order to evaluate the association of persistent lactic acidosis and occult hypoperfusion this study aimed to investigate the following research questions in a standardized porcine polytrauma model: What is the effect of OH on the local circulation of the injured soft tissue in polytrauma? Is OH associated with the amount of blood loss during hemorrhagic shock? How is the local inflammatory reaction of the soft tissue affected by OH?

## Methods

### Study Design

This experimental translational study is based on a previously described standardized porcine polytrauma model (7). Reporting of the results adheres to the ARRIVE Guideline (8).

### Ethical Statement and Housing

Animal housing and experimental protocols were approved by the Cantonal Veterinary Office, Zurich, Switzerland, under license no. ZH 138/2017, and were executed in accordance with Swiss Animal Protection Law, following the “The Principles of Laboratory Animal Care”. Housing and experimental procedures also conformed to the European Directive 2010/63/EU of the European Parliament and of the Council on the Protection of vertebrate animals used for scientific purposes (Council of Europe no. 123, Strasbourg 1985) and to the Guide for the Care and Use of Laboratory Animals (Institute of Laboratory Animal Resources, National Research Council, National Academy of Sciences, 2011). Castrated male Swiss landrace pigs from a disease-free barrier breeding facility were housed in ventilated rooms at a constant room temperature of 21 ± 3°C and a relative humidity of 50%, with natural daylight. Pigs acclimatize to their surroundings for a minimum of 7 days prior to the experiment. takers. Pigs were fasted for a period of 12 h prior to the experiment, water was available ad libitum.

### Animal Care and Monitoring

All pigs were sedated with an intramuscular injection of ketamine (Ketasol^®^-100 ad us.vet.; Dr. E. Graeub AG, Berne, Switzerland; 15 mg/kg body weight), midazolam (Dormicum^®^.; Roche Pharma AG, Basel, Switzerland; 0.5 mg/kg body weight) and atropine (Atropinsulfat KA vet 0.1%; Kantonsapotheke, Switzerland; 0.05 mg/kg body weight). Anesthesia was induced by an intravenous administration of propofol (Propofol ^®^- Lipuro 1%, B. Braun Medical AG; Sempach, Switzerland; 1-2 mg/kg body weight) to achieve relaxation and swallow-reflex diminishment sufficient for intubation. Anesthesia was maintained during the duration of the study with propofol (5-10 mg/kg/h).and Sufentanil (1 μg/kg per hour) under positive pressure ventilation with an FiO2 of 0.3. Following our institutional veterinarian protocol, ventilation was set to maintain an end tidal CO2 (etCO2) of 45-55 mmHg All animals received fluid substitution of 21 ml/h Ringerfundin. Animals were placed in supine position for percutaneous ultrasound guided catheterization of the following vessels: left external jugular vein (6F, Avanti^®^, Cordis^®^ Corporation, Miami Lakes, FL, USA), right femoral artery (5F, Avanti^®^, Cordis^®^ Corporation, Miami Lakes, FL, USA) and left femoral vein (HighFlow Dolphin Catheter, 13F, Baxter International, Deerfield IL, USA). Oxygenation and ventilation parameters, inspirational oxygen fraction, and expiratory oxygen fraction, etCO2, positive end-expiratory pressure (PEEP), respiratory frequency, and tidal volume were continuously monitored. Blood gas analysis was performed to regularly assess pressure of oxygen (paO2) and carbon dioxide (paCO2), Arterial blood pressure and central venous pressure (CVP), as well as heart rate was continuously recorded. A suprapubic catheter was placed in the urinary bladder and diuresis was monitored. During the entire experiment, at least two veterinarians, specialized in anesthesiology were present and monitored the narcosis.

### Induction of Trauma and Resuscitation

Trauma was induced and resuscitation procedures performed, based on a well-established, previously described standardized large animal protocol (7). Briefly, the polytrauma model included a standardized left-sided femoral shaft fracture, a right sided blunt thoracic trauma, a standardized liver laceration, and mean arterial pressure (MAP)-controlled hemorrhagic shock as follows:

Fractures and associated soft tissue injuries were induced by a bolt gun (Blitz-Kerner, turbocut JOBB GmbH, Germany) with cartridges (9x17; DynamitNobel AG; Troisdorf, Germany) to a custom made metal chisel which was placed on the mid of the left femur shaft. The fracture was verified *via* fluoroscopy. The blunt chest trauma was introduced with the same bolt gun on the right lateral thorax wall utilizing an additional lead pael (1.0cm thickness) between the chest and the bolt gun. A chest x-ray was performed and in case of pneumothorax a chest tube was inserted in the usual manner. The liver laceration was performed with a cross-like incision through one third of the liver tissue following median laparotomy. Liver packing was performed following 30 seconds of uncontrolled bleeding. Afterwards the animals underwent MAP-controlled hemorrhagic shock for 60 min until they reached a MAP of 25 ± 5 mmHg. During trauma and hemorrhagic shock, FiO2 was reduced to 0.21 and fluid substitution was lowered to 10ml/h.

One hour after induction of trauma animals were resuscitated following the ATLS, AO/OTA, and AWMF-S3 guideline on Treatment of Patients with Severe and Multiple Injuries^®^) principles (9, 10). Hemorrhagic shock was treated with volume controlled fluid resuscitation (three times the blood loss, Ringerfundin^®^). Normothermia (38.7–39.8°C) was aimed for with warm pads and blankets. Femoral shaft fractures were treated with retrograde intramedullary nailing utilizing a tailored 120 mm nail (cannulated DFN Ø 10.0 mm, DePuySynthes, Raynham, Massachusetts, United States) in the usual manner. The position of the nail was controlled *via* fluoroscopy.

### Measurements of Local Circulation

Local tissue perfusion and microcirculation at the fracture site was assessed using an O2C (Oxygen to see, LEA Medizintechnik GMBH, Giessen, Germany) device. White light spectrometry and laser spectroscopy were used to screen the capillary-venous part of the vascular system (penetration depth of 4–8 mm). Relative local blood flow (flow), flow velocity, local oxygen saturation (sO2) and relative local hemoglobin concentration (rHB) were measured one the vastus lateralis muscle adjacent to the femoral fracture. For this, a 3 cm incision was made on the left lateral thigh and the subcutaneous tissue was exposed by preparation of subcutaneous tissue and incision of the fascia. The measuring probe was placed on the subcutaneous tissue for 1 min above the fracture and measurements were continuously taken every second. Local circulation measurements were further taken at the stomach, and the colon. Data are presented in arbitrary units (A.U.). The reason for the introduction of “Arbitrary Units” is based on the origin of the values. The measured signals are electrical values of frequencies and amplitudes, so that the unit would be a combination of electrical units. Therefore usually a new unit for blood flow is introduced. To calculate the blood flow in ml/min, it would be necessary to compare the electrical signals with a method that measures the blood flow in ml/min (e.g. plethysmography, microspheres) for each organ (or organs with similar optical properties). Then the arbitrary units can be converted in ml/min. This “calibration” has to be done at the measured organ, as there is no artificial model at the moment that simulates tissue in a realistic way.

### Local Inflammation

Local inflammation was measured 6 hours after induction of trauma. Quantikine porcine immunoassay kits from R&D Systems (Minneapolis, MN) were used for analyses of IL-6, IL-8, and IL-10 according to the manufacturer’s protocol. Prior termination of the experiment local soft tissue including fatty tissue and muscle tissue were taken at the fracture site and stored in RNAlater solution (RNAlater Solutions for RNA Stabilization and Storage, ThermoFisher). After shredding of the tissue, RNA extraction was performed using the RNeasy Mini Kit (Qiagen, Hombrechtikon, Switzerland) according to the manufacturer’s protocol. PCR analysis for HSP70, IL-6, IL-8, and IL-10 was performed using the following primer (F forward, R reverse): IL-6 F: 5-GAATCCAGACAAAGCCACCA-3, R: 5-GTGCCCCAGCTACATTATCC-3; IL-8/CXCL8, F: 5-CTTCCAAACTGGCTGTTGCC-3, R: 5-GTTGTTGTTGCTTCTCAGTTCTCT-3; IL-10, F: 5-CGGCGCTGTCATCAATTTCT-3, R: 5-CGGGAACCTTGGAGCAGATT-3; HSP70, F: 5-GCCCTGAATCCGCAGAATA-3, R: 5-TCCCC ACGGTAGGAAACG-3.

### Statistical Methods and Stratification of Animals

Continuous variables are presented with mean and standard deviation (SD), or standard error of the mean (SEM) in graphical presentation, categorical variables as count and percentage. Experimental subjects were stratified according to persistent lactic acidosis as defined by persistent elevated serum lactate level of >2mmol/l (Group OH) despite responding vital signs to resuscitation during the entire observation time. Distribution of data was visualized with histogram and qq-plots. Group comparison on normally distributed continuous variables were performed using the student’s t-test, for non-normal distributed variables the Mann-Whitney U test was utilized; for comparisons on categorical variables the Fisher exact test was used. Adjustment for multiple testing with a false discovery rate at 1% was performed with the Benjamini-Hochberg procedure. The p-value reported is the adjusted value. All statistical analyses were performed using R (R Core Team (2021). R: A language and environment for statistical computing. R Foundation for Statistical Computing, Vienna, Austria. URL https://www.R-project.org/.). GraphPad Prism version 9 for macOS was utilized to print the graphs (GraphPad Software, San Diego, California USA, www.graphpad.com).

## Results

**Figure 1:**
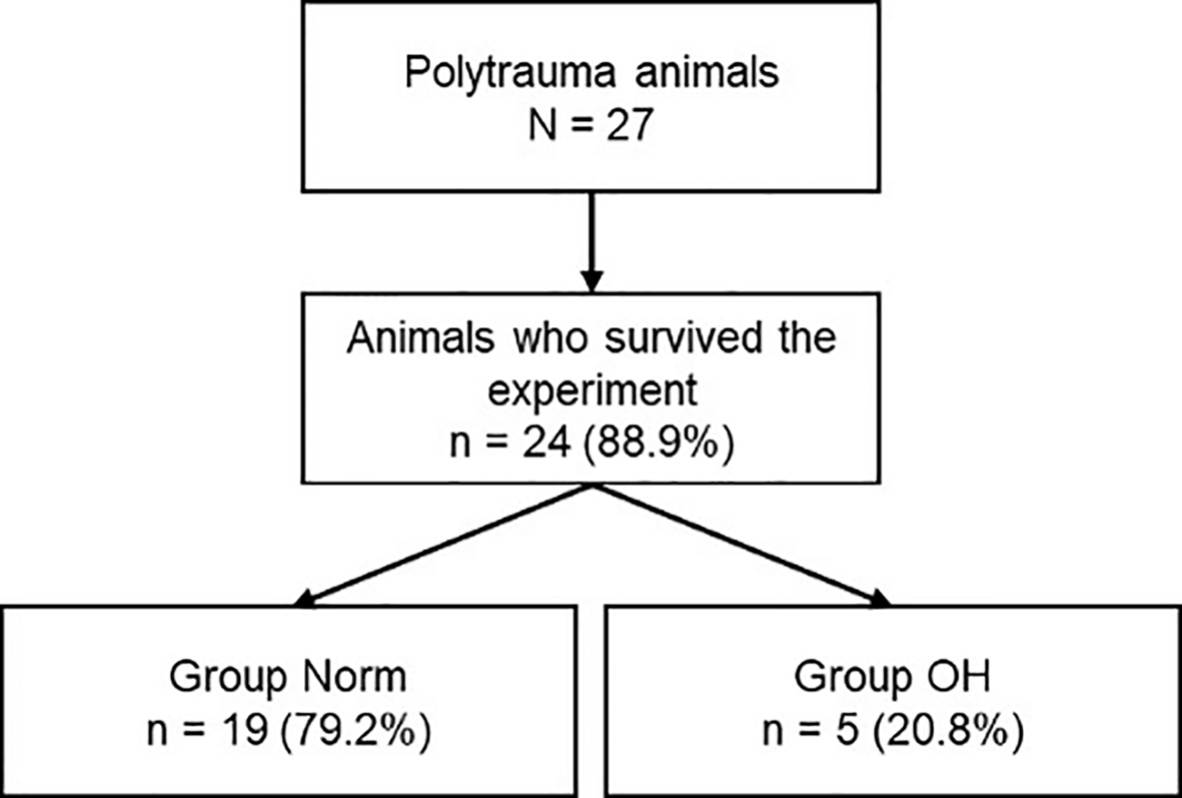
Flowchart and stratification according to occult hypoperfusion (OH) as defined by persistent elevated lactate levels despite responding vital parameters following resuscitation.

This experiment included 27 animals. Out of these 24 (88.9%) survived the experiment and were stratified into Group Norm (n=19, 79.2%) and Group OH (n= 5, 20.8%, **Figure 1**). 3 animals died prematurely and were excluded from analyses.

### Baseline Values

The animals had a mean weight of 50.7kg (SD 4.5kg) representing adolescent animals. At baseline the animals had a MAP of 73.3mmHg (SD 14.7mmHg), a heart rate of 91.5bpm (SD 17.0bpm), and a CVP of 10.1mmHg (SD 3.7mmHg). Baseline lactate level was 1.1mmol/l (SD 0.6mmol/l), base excess 6.3mmol/l (SD 2.4mmol/l), and the temperature 37.0°C (SD 0.7°C). The baseline hemoglobin was 9.7g/dl (SD 0.9g/dl), and the calcium 1.34mg/dl (SD 0.06mg/dl). The animals were comparable in baseline values and characteristics (**Table 1**).

**Table 1:** Baseline characteristics of study animals.

	Group Norm	Group OH	p-value
	19	5	
MAP [mmHg], mean (SD)	72.91 (15.79)	75.20 (9.50)	0.76
Heart rate [bpm], mean (SD)	93.73 (22.02)	88.80 (14.57)	0.64
CVP [mmHg], mean (SD)	10.11 (4.20)	12.40 (4.98)	0.306
Temperature [°C], mean (SD)	36.82 (0.75)	36.62 (0.70)	0.588
pO2 [kPa], mean (SD)	16.95 (4.05)	13.69 (4.09)	0.117
pCO2 [kPa], mean (SD)	5.86 (0.76)	5.51 (0.72)	0.356
pH, mean (SD)	7.45 (0.05)	7.48 (0.04)	0.192
Lactate [mmol/l], mean (SD)	1.14 (0.60)	0.79 (0.17)	0.217
Local blood flow at extremity [AU], mean (SD)	82.51 (22.35)	81.36 (39.37)	0.936
Local sO2 at extremity [AU], mean (SD)	94.45 (7.46)	93.00 (9.43)	0.737
Local rHb amount at extremity [AU], mean (SD)	68.54 (8.56)	74.60 (13.37)	0.255

MAP, Mean arterial pressure; bpm, beats per minute; CVP, central venous pressure; AU, Arbitrary unit; rHb, relative hemoglobin amount; SD, Standard deviation; p-value after Benjamini-Hochberg adjustment.

### Severity of Trauma: Course of MAP and Lactate During Trauma and After Resuscitation

During the hemorrhagic shock, the animals lost a mean of 991.8ml (SD 303.8ml) of blood, and had a MAP of 25mmHg (SD 5mmHg) and a mean heart rate of 94.0bpm (SD 18.9bpm). The lactate level increased to 5.9mmol/l (SD 1.4mmol/l), the temperature was 37.4°C (SD 0.6°C).

**Figure 2:**
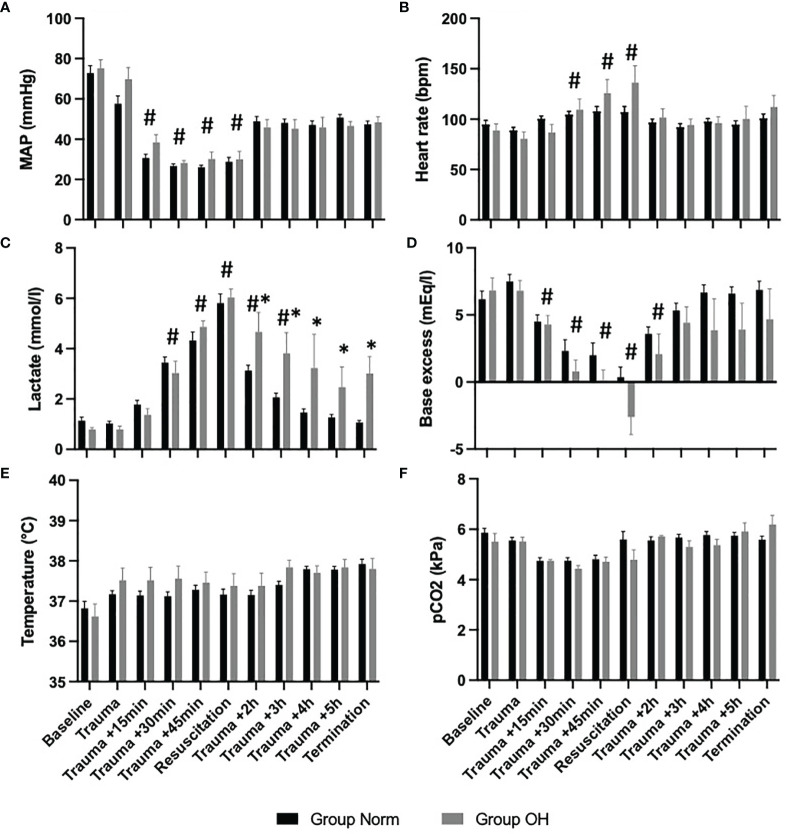
The course of vital parameters, and measures of arterial blood gas analysis during the observational period, presented as mean and standard error of the mean ^#^represents statistical significant difference to baseline, * represents statistical difference among Group Norm and Group OH at the respected timepoint Statistical significance after Benjamini-Hochberg adjustment set at p < 005. **(A)** The course of MAP controlled hemorrhagic shock The MAP decreased significantly compared with baseline values, however, the MAP remained comparable among both groups. **(B)** Significant increase of heart rate (compared with baseline) during hemorrhagic shock and return to comparable values. **(C)** The lactate value increased significantly in both Groups when compared to baseline values After the initiation of resuscitation, the lactate value decreased in Group Norm. in Group OH the lactate value remained significantly higher compared to baseline and to Group Norm. **(D)** Base excess decreased significantly in both groups, and remained lower in Group OH (not statistically significant). **(E)** The Temperature was comparable during the experiment, and the animals were warmed throughout. **(F)** The pCO2 level was utilized for ventilation control and remained comparable during the experiment and among both groups.

Lactate level increased to 6.03mmol/l (SD 0.75mmol/l) in Group OH and remained at a mean of 3.44 mmol/l (SD 1.97mmol/l). The lactate level decreased in Group Norm following resuscitation to baseline values. The temperature remained comparable in both groups throughout the experiment as did the pCO2 levels (**Figure 2**).

### Occult Hypoperfusion Is Associated With Reduced Local Circulation in the Soft Tissue of the Extremity

Local soft tissue circulation measurements on the hind extremities have shown a significantly reduced microcirculation in Group OH when compared with Group Norm, with local SpO2 in the vastus muscle being significantly lower (Group OH: 60.4, SD 4.6 A.U. versus Group Norm 75.8 SD 12.8 A.U., p=0.049). Local Hb values were also significantly reduced in the OH group (Group OH: 39.4, SD 5.3 A.U. versus Group Norm 63.9, SD 27.6 A.U., p=0.031). No differences were found between the groups in the microcirculation of the abdominal organs, such as the stomach, colon or liver (**Table 2**).

**Table 2:** Occult hypoperfusion is associated with reduced local circulation in injured soft tissue 6 hours after trauma.

	Group Norm	Group OH	p-value
Local blood flow at extremity [AU], mean (SD)	75.75 (12.79)	60.38 (4.56)	0.049
Local rHb amount at extremity [AU], mean (SD)	63.92 (27.63)	39.39 (5.33)	0.031
Local sO2 at extremity [AU], mean (SD)	72.02 (6.13)	72.09 (7.84)	0.987
Local blood flow at stomach [AU], mean (SD)	176.23 (14.9)	153.58 (51.3)	0.159
Local rHb amount at stomach [AU], mean (SD)	52.28 (24.08)	58.80 (21.34)	0.674
Local sO2 at stomach [AU], mean (SD)	72.02 (6.13))	72.09 (7.84)	0.987
Local blood flow at colon [AU], mean (SD)	174.66 (34.08)	166.03 (30.51)	0.689
Local rHb amount at colon [AU], mean (SD)	67.02 (4.98)	64.42 (3.89)	0.887
Local sO2 at colon [AU], mean (SD)	53.36 (17.41)	53.60 (18.20)	0.983

AU, Arbitrary units; SD, standard deviation; OH, occult hypoperfusion; p-value after Benjamini-Hochberg adjustment.

### Occult Hypoperfusion Is Associated With an Increased Local Inflammatory Response

The analysis of local soft tissue inflammation revealed a significantly higher concentration of pro-inflammatory cytokines in animals in Group OH when compared with Group Norm. Interleukin-6 was approximately 3 times higher in the OH group when compared to the Norm group (Group OH: 318.3, SD 326.6 pg/ml versus Group Norm 73.9. SD 96.3 pg/ml, p=0.03). Similarly, IL-8 has shown 4 times higher values (Group OH: 442.9, SD 687.6 pg/ml versus Group Norm 72.3, SD121.3 pg/ml, p=0.056). Interleukin-10 and HSP were comparable between the groups (**Table 3**).

**Table 3:** Occult hypoperfusion is associated with increased local inflammatory response of the injured soft tissue at the fracture.

	Group Norm	Group OH	p-value
HSP fatty tissue [pg/ml], mean (SD)	4.93 (12.68)	5.88 (9.79)	0.892
IL8 fatty tissue[pg/ml], mean (SD)	72.33 (121.31)	442.94 (687.55)	0.056
IL6 fatty tissue [pg/ml], mean (SD)	73.97 (96.29)	318.30 (326.56)	0.03
IL10 fatty tissue [pg/ml], mean (SD)	4.81 (4.29)	3.23 (2.78)	0.558
IL6 muscle [pg/ml], mean (SD)	69215.23 (86342.31)	76003.88 (131083.13)	0.908
IL10 muscle [pg/ml], mean (SD)	1907.29 (3432.24)	286.99 (369.85)	0.435

HSP, Heat shock protein; IL, interleukin; SD, standard deviation; OH, occult hypoperfusion; p-value after Benjamini-Hochberg adjustment.

## Discussion

The aim of this study was to analyze the association of OH on the local microcirculation and the local inflammatory of the injured soft tissue reaction following a standardized porcine polytrauma model and found the following points:

• Persistent elevated lactate levels can be present despite standardized trauma and resuscitation protocols.
• Animals in Group OH show reduced local circulation at the injured extremity when compared with Group Norm.
• The local inflammatory reaction of the soft tissue in the injured extremity is significantly higher Group OH when compared with Group Norm.

The identification of multiple injured patients with insufficient response to resuscitation is required to choose an appropriate treatment strategy. Studies have reported a negative outcomes in patients with persistent occult hypoperfusion (OH) (11). There still is, however, a lack of consensus on the definition of OH: Some studies defined OH based on the pH level (pH < 7.25) (12) others described significant hemorrhage despite hemodynamic stability to be associated with occult hypoperfusion (13). A base deficit of less than -4.5mmol/l was further a proposed threshold for OH (14). However, most articles define occult hypoperfusion based on persistent elevated lactate levels, despite normal vital signs (15–17). The range of the cut-off for persistent lactic acidosis varies from 2mmol/l (18), to 2.4 mmol/l (19), 2.5 mmol/l (16), and 3mmol/l (20). The present study has chosen the cut-off of 2mmol/l since this value is clinically one relevant factor in distinguishing between stable and unstable trauma patients (1, 2). This is the first study that provides some evidence for the development of OH despite standardized trauma and standardized treatment protocols. The pathophysiologic cause for OH remains subject of investigations and is hypothesized to be the result of multifactorial pathways. If the oxygen supply decreases other mechanisms attempt to restore oxygenation (21). Hypoxia can induced inflammation and vice versa (22). This association is based on experimental designs that assessed toll-like receptors (TLRs) in kidney or lung transplantation, and the resulting insulin resistance following macrophage migration in adipose tissue (22). The present study provides new data that shows the association of OH with increased inflammation in acute injured soft tissue in a polytrauma model. Ischemia and reperfusion injuries result in sterile inflammation following activation of TLRs, recruitment and activation of immune cells of the innate and adaptive immune system and activation of the complement system (23). This clinically very relevant association has been discussed following biomolecular and histopathologic studies. The present investigation provides a translational approach that introduces standardized quantification methods for OH and local hypoperfusion that might be feasible for routine clinical use. Changes in local circulation depend further on the specific organ of interest (24). The relevance of ischemia-reperfusion injury is further associated with local circulation and represents a challenging condition in trauma patients (25). It has further been shown that the local soft tissue injury serves as a relevant factor for the prediction of mortality in severely injured patients (1, 2). OH and altered microcirculation might be utilized to quantify the severity of the local soft tissue damage. OH might further be associated with persistent centralization. The present study showed that the abdominal organs are not as severely affected by circulatory changes during OH when compared with the extremity. The centralization appears to persist during OH and is an increased risk for complications and mortality (26).

Shock and resuscitation have a substantial impact on the local circulation and the local inflammation (27). Systemic inflammatory response is associated with the severity of trauma and skeletal muscle oxygenation and represents a relevant factor for resuscitation strategies and success (28). The release of damage-associated molecular patterns (DAMPs) following trauma are associated with increased inflammation. Systemic inflammation is increased in polytrauma and the damaged local soft tissue might be responsible for the inflammatory reaction (29). The elevated local inflammation impairs the coagulatory system, increases vascular leakage by damaging endothelium and might therefore subsequently damage remote organs (30).

The presented results are in accordance with previously published data that have shown an adequate response to resuscitation within 30 minutes (31). The local circulatory flow rate correlates with the MAP, and following hemorrhagic shock rHB and local O2 measures decreased significantly (27). These data are comparable to the present experiment that shows an effect of the trauma and the hemorrhage to the local circulation. But the present experiment goes one step further, by providing data on an even further effect of OH on the local circulation and inflammation. The systemic response to severe trauma has been shown (31), the effect of polytrauma on local circulation can be quantified (27), and following our results, the severity of decreased local circulation or increased local inflammation of injured soft tissue is further associated with OH.

Experimental studies allow detection of these markers, however, the transfer to human situation remains challenging (32). The present study allows the association of OH, decreased local circulation, and increased local inflammation and might therefore support the translation of these results to the human situation. The quantification of the local tissue injury is relevant for the risk for complications and for the treatment strategy (33). The local inflammatory response is time-dependent and therefore provides an association of OH and increased local inflammation (34).

### Strengths and Limitations

This study is conducted as an experimental study. The strengths of this study are the standardized induction of trauma, and the standardized resuscitation methods. The included study subjects were comparable in baseline characteristics and the observation time were standardized. These conditions are rarely found in clinical trials. OH might be the result of the local microcirculation. Following the present study, we provide data that show a certain association of OH and local microcirculation. Further, this association was measured in a standardized and reproducible manner. However, the reported data cannot prove a cause-relation effect that provides evidence whether the decreased local circulation causes OH or vice versa. Based on the standardized trauma and standardized resuscitation the effect should be investigated on an epigenetic level, since it might be based on an individual reaction to the injury. One might argue that the translation of our results to routine clinical practice might be lacking, however, large animals mirror human physiology better when compared with rodents (35). For those reasons, a certain translation of our results might be feasible. This experiment was designed to analyze the short term effect of trauma on inflammation and circulation. Therefore, long term complications, infections, or mortality were not observed. Albeit this shortcoming, we believe that the presented results might improve the pathophysiologic understanding of OH and its association with decreased local circulation at the extremity and increased local inflammation.

## Conclusion

OH is associated with decreased local perfusion and increased local inflammation at the injured soft tissue and might serve as a measure for severity of local soft tissue injuries, guide treatment strategies, and measure treatment success.

## Data Availability Statement

The raw data supporting the conclusions of this article will be made available by the authors, without undue reservation.

## Ethics Statement

Animal housing and experimental protocols were reviewed and approved by the Cantonal Veterinary Office, Zurich, Switzerland, under license no. ZH 138/2017, and were executed in accordance with Swiss Animal Protection Law, following the “The Principles of Laboratory Animal Care”. Housing and experimental procedures also conformed to the European Directive 2010/63/EU of the European Parliament and of the Council on the Protection of vertebrate animals used for scientific purposes (Council of Europe no. 123, Strasbourg 1985) and to the Guide for the Care and Use of Laboratory Animals (Institute of Laboratory Animal Resources, National Research Council, National Academy of Sciences, 2011).

## Author Contributions

SH and YK contributed equally to this work, conducted the research, collected the data, analysed and interpreted the data, wrote the original draft and critically reviewed the manuscript. MPJT performed the study, was involved in funding acquisition, collected data, interpreted the data and critically reviewed the manuscript. HT interpreted the data, critically reviewed the manuscript. MW and NC conducted the study, performed the anaesthesia on animals, were in charge of housing and animal welfare, collected the data, critically reviewed the manuscript. H-CP and RP supervised the study, were involved in funding acquisition, critically reviewed the manuscript. RP conducted the study, supervised data collection, interpreted the data, developed the study idea, wrote the original draft critically reviewed the manuscript.

## Funding

This study was supported by the AO Grant S-16–133T: Effects of standard reaming and RIA techniques on local soft tissue and systemic homeostasis in a porcine trauma model.

## Conflict of Interest

The authors declare that the research was conducted in the absence of any commercial or financial relationships that could be construed as a potential conflict of interest.

## Publisher’s Note

All claims expressed in this article are solely those of the authors and do not necessarily represent those of their affiliated organizations, or those of the publisher, the editors and the reviewers. Any product that may be evaluated in this article, or claim that may be made by its manufacturer, is not guaranteed or endorsed by the publisher.
